# Different mechanisms activated by mild versus strong loading in rat Achilles tendon healing

**DOI:** 10.1371/journal.pone.0201211

**Published:** 2018-07-25

**Authors:** Malin Hammerman, Franciele Dietrich-Zagonel, Parmis Blomgran, Pernilla Eliasson, Per Aspenberg

**Affiliations:** Experimental orthopedics, Department of Clinical and Experimental Medicine, Faculty of Health Science, Linköping University, Linköping, Sweden; University of Pittsburgh, UNITED STATES

## Abstract

**Background:**

Mechanical loading stimulates Achilles tendon healing. However, various degrees of loading appear to have different effects on the mechanical properties of the healing tendon, and strong loading might create microdamage in the tissue. This suggests that different mechanisms might be activated depending on the magnitude of loading. The aim of this study was to investigate these mechanisms further.

**Methods:**

Female rats had their right Achilles tendon cut transversely and divided into three groups: 1) unloading (calf muscle paralysis by Botox injections, combined with joint fixation by a steel-orthosis), 2) mild loading (Botox only), 3) strong loading (free cage activity). Gene expression was analyzed by PCR, 5 days post-injury, and mechanical testing 8 days post-injury. The occurrence of microdamage was analyzed 3, 5, or 14 days post-injury, by measuring leakage of injected fluorescence-labelled albumin in the healing tendon tissue.

**Results:**

Peak force, peak stress, and elastic modulus of the healing tendons gradually improved with increased loading as well as the expression of extracellular matrix genes. In contrast, only strong loading increased transverse area and affected inflammation genes. Strong loading led to higher fluorescence (as a sign of microdamage) compared to mild loading at 3 and 5 days post-injury, but not at 14 days.

**Discussion:**

Our results show that strong loading improves both the quality and quantity of the healing tendon, while mild loading only improves the quality. Strong loading also induces microdamage and alters the inflammatory response. This suggests that mild loading exert its effect via mechanotransduction mechanisms, while strong loading exert its effect both via mechanotransduction and the creation of microdamage.

**Conclusion:**

In conclusion, mild loading is enough to increase the quality of the healing tendon without inducing microdamage and alter the inflammation in the tissue. This supports the general conception that early mobilization of a ruptured tendon in patients is advantageous.

## Introduction

Ruptures of the Achilles tendon are common, and rehabilitation takes a long time [1]. The treatment usually includes immobilization in a brace, i.e. a cast or an orthosis [2]. However, animal studies have shown that early mechanical loading improves tendon healing [3–7]. As little as 5 minutes of loading each day can increase the strength of the healing tendon in rats and also alter the gene expression profile [8, 9]. Many clinics have developed routines for early mobilization after Achilles tendon rupture, and report favorable results [2, 10]. These rehabilitation programs includes mild mechanical loading of the healing tendon while most animal studies investigates the effect of strong mechanical loading [2, 3, 5, 7, 10–12]. We have recently shown that mild and strong loading seems to have different effects on strength, quality, and size of the healing tendon and might activate different mechanisms [3].

It is assumed that the response to loading during tendon healing is mainly exerted via mechanotransduction mechanisms similar to intact tendons [13, 14]. In other words, the cells sense the forces during loading and respond to this by, for example increasing collagen expression [13]. However, the healing tendon early after rupture is weak, and strong loading might create microdamage in the tissue which probably affects the inflammatory response [5]. Indeed, strong loading (by treadmill walking in a rat model) during early tendon healing has been shown to increase the expression of genes involved in the inflammatory response [6, 9]. Strong loading (by free cage activity) has also been shown to affect the immune cell composition by prolonging the pro-inflammatory response and delaying the switch to an anti-inflammatory response in the healing tissue [15]. Furthermore, microdamage induced by needle penetration, appears to stimulate tendon repair in a similar way as loading [5, 16]. These results indicate that the stimulatory effect of strong loading can, at least in part, be exerted by trauma leading to microdamage and an altered inflammatory response.

The aim of this study was to clarify the mechanisms activated during different degrees of loading in healing tendons. We studied mechanical properties and gene expression in rat healing tendons exposed to unloading, mild loading or strong loading. The gene expression analysis focused on genes involved in extracellular matrix production and inflammation. The mechanical properties were examined 8 days post-injury, and the gene expression was analyzed after 5 days in order to leave some time for gene expression changes to influence the tissue and its mechanics. We also wanted to verify the occurrence of microdamage after strong mechanical loading, by measuring fluorescent protein leakage, as a sign of blood leakage in the tissue. This was tested in different phases of tendon healing, i.e. the inflammatory (day 3), the proliferative (day 5), and the remodeling phase (day 14).

We hypothesized that mild mechanical loading would increase the structural and material properties of the healing tendon tissue, and that strong mechanical loading would create microdamage, an altered inflammatory response, and ultimately a larger tendon size. We also hypothesized that fluorescent protein leakage (as a sign of microdamage) would be increased after strong loading compared to mild loading when the tendon is weak (at day 3 and 5), but not when it is stronger (at day 14).

## Materials and methods

### Study design

164 Sprague-Dawley female rats were used (6–8 weeks old). The right Achilles tendon was transversely cut in all rats. The rats were used to: 1) analyze the effect of loading on mechanical properties, 2) analyze the effect of loading on gene expression, and 3) verify microdamage as a result of strong loading.

66 rats were used for analysis of 3 different loading conditions. They were randomly assigned into 3 groups: 1) unloading (paralysis of the calf muscles, using Botox injections, combined with joint fixation with a steel-orthosis), 2) mild loading (Botox injections only), and 3) strong loading (free cage activity). The rats were euthanized 5 days after tendon injury for gene expression analysis (N = 12) and after 8 days for analysis of mechanical properties (N = 10).

98 rats were used to verify microdamage by measuring leakage of fluorescent labeled bovine serum albumin into the tissue. The rats were randomly assigned to mild or strong loading, and euthanized 3, 5 or 14 days after tendon injury (N = 12).

All experiments were approved by the local ethical committee (Regional Ethics Committee for animal experiments in Linköping, ref 15–15), and adhered to institutional guidelines. All surgery and Botox injections were performed under anesthesia with isoflurane gas, and all efforts were made to minimize suffering. All rats were randomized by lottery and all evaluations were performed blinded from group belonging. Four rats in the gene expression study were excluded because of RNA extraction failure.

### Botox injections

Rats were given intramuscular Botox injections to induce plantar flexor muscle paralysis 3 days before tendon transection. The rats were anesthetized with isoflurane gas (Forene, Abbot Scandinavia, Solna, Sweden). Botulinum toxin (Botox, Allergan, Irvine, CA) was injected into the gastrocnemius lateralis, medialis and the soleus muscles at a dose of 1 U/muscle, giving a total dose of 3 U/animal and a total volume of 0.06 mL. The animals were thereafter allowed free cage activity.

### Surgery

All rats were anesthetized with isoflurane gas and given antibiotics (25 mg/kg, Oxytetracycline, Engemycin; Intervet, Boxmeer, The Netherlands) preoperatively. They were also given analgesics (0.045 mg/kg Buprenorphine, Temgesic; Schering-Plough, Brussels, Belgium) preoperatively and regularly until 48 hours after surgery. The surgery was performed under aseptic conditions. A transverse skin incision was made lateral to the Achilles tendon, and the tendon complex was exposed. The plantaris tendon was removed. The Achilles tendon was cut transversely, and a 3 mm-segment was removed. The tendon was left to heal spontaneously without sutures. The skin was closed by two stitches.

### Unloading with a steel-orthosis

**Fig 1:**
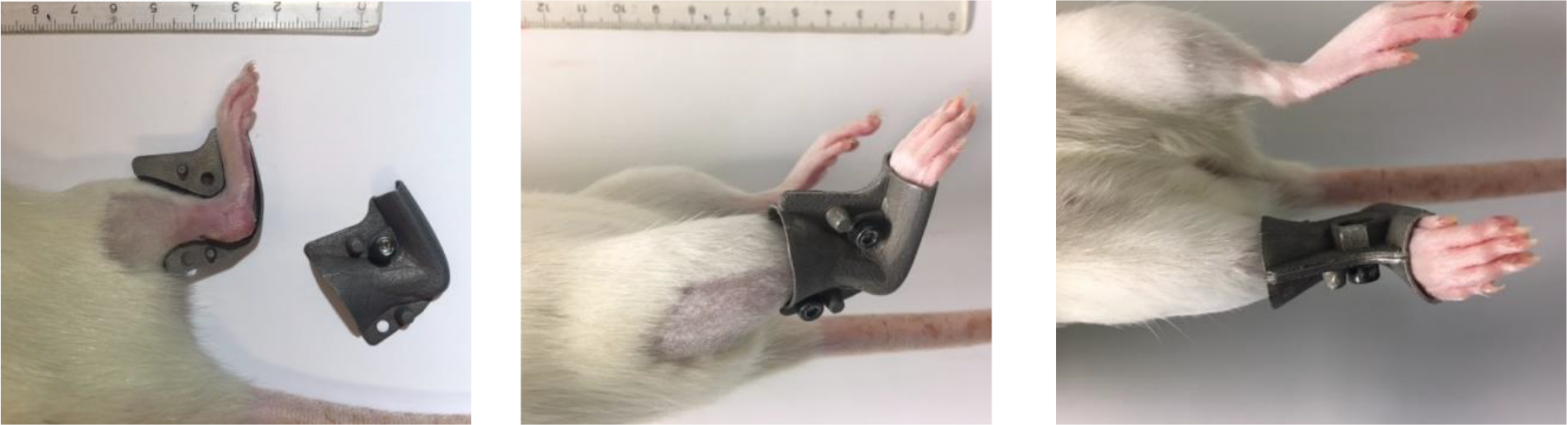
Unloading device. Steel-orthosis, used in the unloaded group.

A new method to achieve complete unloading was applied in this study, using a combination of Botox for muscle paralysis and a customized orthosis for rats made from steel (made by Prodelox, Linköping, Sweden) to prevent joint movement and passive loading (Fig 1). Botox-injected rats received an orthosis made by steel on their right hind limb directly after surgery. The steel-orthosis was made of 2 parts held together by 2 screws, and weighted 15.9 gram. It was kept on during the whole time until euthanasia.

### Mechanical evaluation

The rats used for mechanical evaluation were euthanized with carbon dioxide. The right Achilles tendon, together with the calcaneal bone and the gastrocnemius and soleus muscle complex, was dissected free from the skin and harvested. Sagittal and transverse diameter of the healing tendon tissue were measured with a slide caliper, and transverse area was calculated by assuming an elliptical geometry (sagittal *d* * transverse *d* * π)/4). The distance between the old tendon stumps (the gap distance) was measured, by visualization through the partly transparent healing tissue. The muscles were carefully scraped off from the tendon fibers, and the fibers were fixed in a metal clamp by fine sandpaper. The bone was fixed in a custom-made clamp, at 30° dorsiflexion relative to the direction of traction, in the material testing machine (100R, DDL, Eden Prairie, MN). The machine pulled at a constant speed of 0.1 mm/s until failure and location of failure was recorded for each sample. Peak force at failure (N), stiffness (N/mm), and energy uptake (Nmm) were calculated by the software of the testing machine. The investigator marked a linear portion of the elastic phase of the curve for stiffness calculation. Peak stress (peak force/transverse area) and elastic modulus ((stiffness*gap distance)/transverse area) were calculated afterwards, assuming an elliptic cylindrical shape and homogenous mechanical properties. All measurements and calculations were carried out by a blinded investigator.

### RNA extraction

Rats used for gene expression analysis were anesthetized with isoflurane gas. The skin was shaved and washed with chlorhexidine (Fresenius Kabi, Sweden), and the healing tissue was dissected free from the skin and the surrounding soft tissue under aseptic conditions. A segment from the healing Achilles tendon (consisting of newly formed tissue only) was harvested, quickly rinsed in sodium chloride, snap-frozen in liquid nitrogen, and stored at -70°C until RNA extraction. Thereafter, the rats were euthanized with an overdose of pentobarbital sodium. A combination of the Trizol method and RNeasy total mini kit (QIAGEN, Sollentuna, Sweden) was used to extract RNA. The tendons were frozen in liquid nitrogen and were pulverized one by one by a tungsten ball in cooled vessels by a Retsch mixer mill MM 200 (Retsch, Haan, Germany). Trizol was added to the pulverized tendons and was thawed at room temperature. Thereafter, chloroform was added, followed by centrifugation and phase separation. The aqueous phase of the samples was transferred to new tubes containing 70% ethanol. The RNeasy total mini kit was used, according to the manufacturers’ instructions, to further purify RNA. DNase treatment was used to eliminate potential DNA contamination. RNA quality and concentration were analyzed with RNA 6000 Nano kit (Agilent Technologies, Böblingen, Germany) and Nanodrop ND-1000 spectrophotometer (NanoDrop Technologies, Wilmington, DE). RNA samples were stored at -70°C.

### Quantitative real-time PCR

1.5 μg of total RNA was transcribed into cDNA using a high-capacity cDNA reverse transcription kit (Applied Biosystems, United Kingdom) and diluted in Tris-EDTA buffer. Gene expression changes were measured for genes related to inflammation and extracellular matrix. 23 primers were used and purchased from Applied Biosystems (Life Technologies, Netherlands, Table 1). Amplification was performed in 15 μL reactions using TaqMan Fast PCR Master mix (Life Technologies, Great Britain). Data was analyzed using the ΔΔC_t_ method and each sample was normalized to *Ubiquitin C* [17]. The efficiency for the reference gene *Ubc* and the target genes were all acceptable, with a slope of ≤ 0.1, except IFN-γ (0.18). The expression of the reference gene did not vary significantly between the groups. Reactions with no reverse transcription and no template were added as negative controls. The plate efficiency was controlled using a mixture of all samples systemically distributed in 6 wells of each plate. The down-regulated genes had a fold change between 0 and 1 and were converted to a negative value by this formula: -1/fold change.

**Table 1:** Primers used for qRT-PCR (purchased from applied Biosystems).

Inflammation	**Gene symbol**	**Gene name**	**Ref.sequence**	**Assay identification**
	**Pro-inflammatory mediators**			
	*IL-1β*	*Interleukin 1β*	NM_031512.2	Rn00580432_m1
	*TNF*	*Tumor necrosis factor*	NM_012675.3	Rn00562055_m1
	*IFN-γ*	*Interferon γ*	NM_138880.2	Rn00594078_m1
	*IL-6*	*Interleukin 6*	NM_012589.2	Rn01410330_m1
	*PTGS2*	*Cyclooxygenase 2 (COX-2)*	NM_017232.3	Rn01483828_m1
	*PTGES*	*Prostaglandin E synthase*	NM_021583.3	Rn00572047_m1
	*NOS2*	*Nitric oxide synthase (inducible)*	NM_012611.3	Rn00561646_m1
	**Recruitment of leukocytes**			
	*SELE*	*E-selectin*	NM_138879.1	Rn00594072_m1
	*ICAM-1*	*Intercellular adhesion molecule 1*	NM_012967.1	Rn00564227_m1
	*VCAM-1*	*Vascular cell adhesion molecule 1*	NM_012889.1	Rn00563627_m1
	*CXCL1*	*Chemokine (C-X-C motif) ligand 1*	NM_030845.1	Rn00578225_m1
	*CCL2*	*Chemokine (C-C motif) ligand 2*	NM_031530.1	Rn00580555_m1
	*CCL7*	*Chemokine (C-C motif) ligand 7*	NM_001007612.1	Rn01467286_m1
	**Anti-inflammatory mediators**			
	*IL-10*	*Interleukin 10*	NM_012854.2	Rn00563409_m1
Matrix	**Extracellular matrix**			
	COL1A1	Collagen 1	NM_053304.1	Rn01463848_m1
	COL3A1	Collagen 3	NM_032085.1	Rn01437681_m1
	COL5A1	Collagen 5	NM_134452.1	Rn00593170_m1
	LOX	Lysyl oxidase	NM_017061.2	Rn01491829_m1
	ELN	Elastin	NM_012722.1	Rn01499782_m1

### Preparation for microdamage detection

The detection of microdamage is a new method that has been developed for this study. One hour before euthanasia, the rats were anesthetized with isoflurane gas and given an intravenous injection with 1 mL of fluorescent bovine serum albumin (Albumin–fluorescein isothiocyanate conjugate, BSA-FITC, Sigma; 10 mg/mL dissolved in PBS). 15 minutes after the injection, half of the rats (49 rats) were randomized to treadmill walking for 5 minutes (the strong loading group). The treadmill was used to ensure that all rats in the strong loading group were applied to mechanical stimulation after the injection. The treadmill settings were 9 m/min with 7.5° uphill slope. All 98 rats in the microdamage experiment had been habituated to the treadmill before the experiment started. 30 minutes after the injection, all rats were anesthetized with isoflurane gas again. The tip of the tail was cut and blood was collected. The blood was protected from light and centrifuged at 13 400 rpm (12x4 g, eppendorf) for 5 minutes. The blood plasma was stored at -20°C. The anesthetized rats were thereafter shaved on the right leg and abdomen. An incision was made over the whole abdomen and the aorta visualized and ligated. A hole was made in the aorta distal to the ligature and a needle introduced and fixed for limb perfusion of the injured leg. 1 mL of lidocaine (10 mg/mL, FarmaPlus) was first injected followed by 20 mL of saline. During this procedure the vein was opened for drainage. The perfusion was done to remove intravascular blood to ensure that the method would only measure extravasated blood and not increased vascularity. The healing tendon was dissected free from the skin and soft tissue, but still contained the old tendon. The tissue was put in a tube, protected from light, and stored at -70°C. The rats were thereafter killed, under anesthesia, by removal of the heart.

### Detection of microdamage (leakage of BSA-FITC)

Homogenization of the tissue was performed while the tendons were kept frozen in liquid nitrogen and pulverized one by one by a tungsten ball in a cooled vessel in a Retsch mixer mill MM 200 (Retsch, Haan, Germany), 30 Hz for 1 minute. 300 μL PBS was added to each vessel, and after thawing, the liquid was stored at -70°C. BSA-FITC was measured both in the blood plasma and the homogenized tendons. The plasma was thawed and vortexed. 120 μL PBS was mixed with 80 μl plasma in a 96-well plate. The tendon tissue liquid was thawed, vortexed and centrifuged at 13 400 rpm (12x4 g, eppendorf) for 2 minutes. After centrifugation, the tubes contained a pellet (cell debris), a clear water phase and a fatty phase on top. 200 μL of the water phase was added to the 96-well plate and PBS was added if needed to reach 200 μL. Blood plasma and tendon tissue for each experimental day were measured in the same plate. 1 mg/mL BSA-FITC was added as a positive control and PBS was added as a negative control. 96-well black plates were used. The fluorescence was measured with a plate reader (VICTOR X4, PerkinElmer, USA) having excitation at 485 nm and emission at 535 nm. The tubes and the plates were protected from light during the entire experiment. The fluorescence detected from the tendon tissue was corrected if diluted with PBS and normalized to the tissue weight and the fluorescence detected in the blood plasma.

### Evaluation of the microdamage detection method

To evaluate the effect of limb perfusion, 20 rats were randomly divided into two groups; mild loading or strong loading (N = 10). All rats were treated in the same way according to “Preparation for microdamage detection” except that the leg was not perfused with saline to remove intravasal fluorescence and they were euthanized with carbon dioxide.

To evaluate possible auto-fluorescence in the tissue, 6 new rats were randomly divided into two groups; mild loading or strong loading (N = 3). They were treated in the same way according to “Preparation for microdamage detection” except that they were not injected with BSA-FITC.

### Statistics

All data were analyzed in IBM SPSS version 23 and log-transformed [18] before statistical testing to achieve similar variance in the different groups.

For the mechanical parameters, a one-way ANOVA was used (significance level 0.05) followed by Student´s t-test comparing strong loading *vs* mild loading and mild loading *vs* unloading. We refrained from other comparisons, so the cutoff for significance with Bonferroni´s correction was set to 0.025.

For the gene expression data, Student´s t-test was performed comparing strong loading *vs* mild loading and mild loading vs unloading. Because many genes were tested, we could not correct for multiple testing. The p-values (significance level 0.05) in this analysis should therefore be seen as descriptive indicators only.

In order to enable comparisons between mechanical and gene expression variables, also mechanical treatment effects are expressed as fold change, defined as highest group mean/lowest group mean. If this ratio indicates a decrease, it gets a negative value (no change is a fold change of 1, a doubling is described as fold change 2, and a reduction by half as -2).

Each time-point for detection of microdamage was analyzed separately. The two groups, mild vs strong loading, were analyzed with Student´s t-test and significance was set to 0.017 because of multiple comparisons (3 time-points, Bonferroni correction).

To evaluate the effect of limb perfusion, Student´s t-test was performed on tendons with mild loading day 5 (rinsed versus not rinsed). To verify the effect of loading on unrinsed tendons day 5 (mild vs strong loading), Student´s t-test was performed. Significance was set to 0.05 for each analysis.

## Results

### Mechanical testing 8 days post-injury

**Fig 2:**
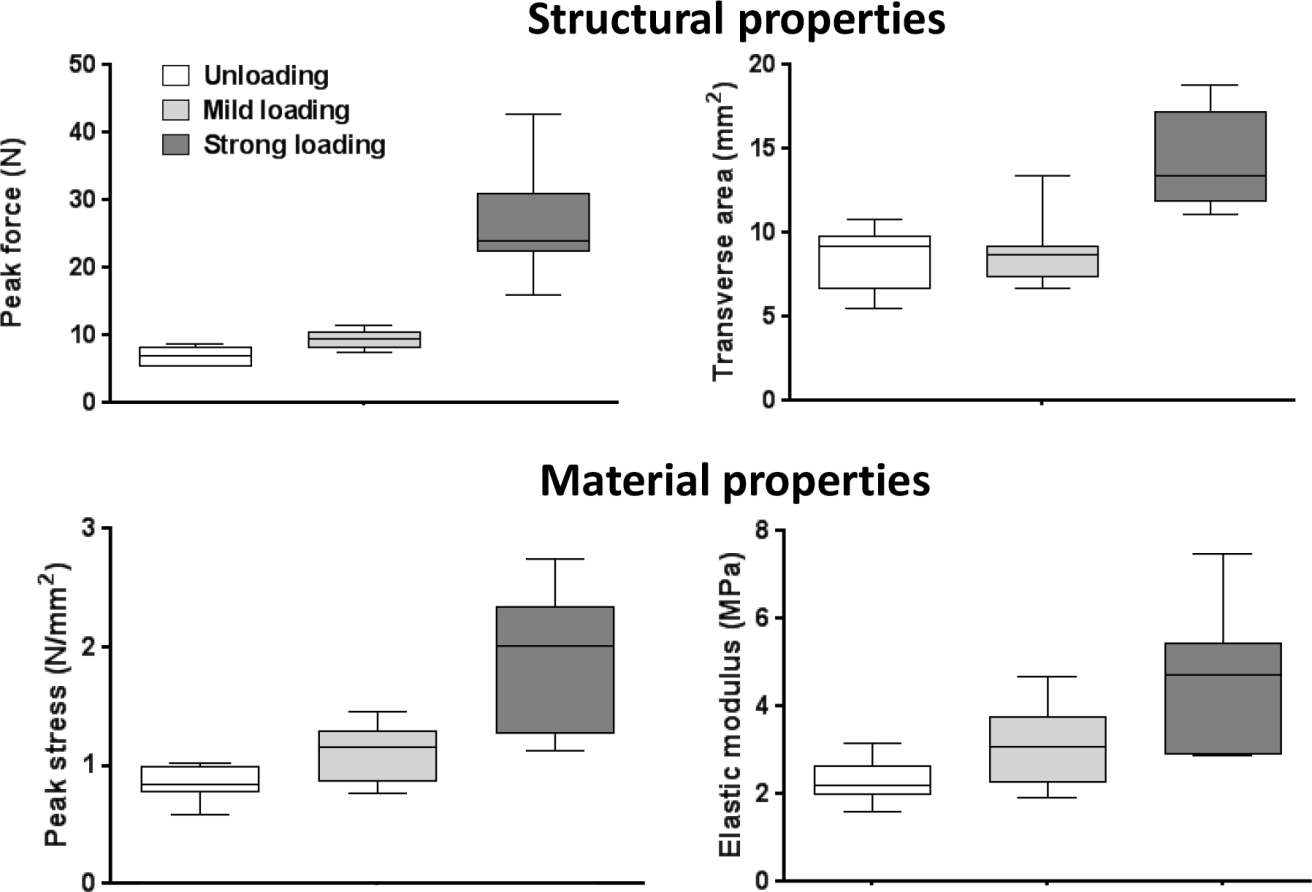
Mechanical testing results. Mechanical testing results for peak force, transverse area, peak stress, and elastic modulus, 8 days after tendon injury. Three different loading conditions were tested: unloading (Botox and steel-orthosis), mild loading (Botox), and strong loading (free cage activity). N = 10.

All samples ruptured in the newly formed tendon tissue. The one-way ANOVA, comparing all three groups, showed significant differences for all mechanical parameters tested (p < 0.05). Compared to unloading, mild mechanical loading increased peak force, stiffness, peak stress, and elastic modulus (p < 0.025; Fig 2, Table 2), but not transverse area. The fold change (defined as mild loading/unloading) for these parameters ranged from 1.3 to 1.6 (Table 2). Compared to mild loading, strong loading increased the values for all parameters (including transverse area) and the fold change ranged from 1.6 to 5.3 (p < 0.025; Fig 2, Table 2, S1 Dataset).

**Table 2:** Mechanical properties of rat Achilles tendons, 8 days post-injury.

	Unloading	Mild loading	Strong loading	From unloading to mild loading		From mild to strong loading	
N	10	10	10	FC	p	FC	p
**Structural properties**							
Gap distance (mm)	**4.05** (0.64)	**4.58** (0.63)	**10.8** (1.75)	1.13	0.072	**2.35**	**< 0.001**
Transverse area (mm^2^)	**8.51** (1.88)	**8.81** (1.88)	**14.3** (2.86)	1.04	0.679	**1.62**	**< 0.001**
Peak force (N)	**7.13** (1.27)	**9.49** (1.34)	**26.4** (7.29)	**1.33**	**0.001**	**2.78**	**< 0.001**
Stiffness (N/mm)	**1.67** (0.32)	**2.38** (0.45)	**3.91** (0.89)	**1.42**	**0.001**	**1.64**	**< 0.001**
Energy (Nmm)	**10.4** (2.89)	**11.9** (2.59)	**63.4** (20.3)	1.15	0.197	**5.30**	**< 0.001**
**Material properties**							
Peak stress (MPa)	**0.86** (0.14)	**1.11** (0.24)	**1.90** (0.56)	**1.30**	**0.011**	**1.71**	**< 0.001**
Elastic modulus (MPa)	**2.27** (0.45)	**3.01** (0.89)	**4.62** (1.45)	**1.59**	**0.007**	**2.35**	**< 0.001**

Values are mean ± SD. FC means fold change, and are defined as highest group mean/lowest group mean. p-values are from Student´s t-test with a significant cutoff at 0.025 (Bonferroni´s correction). N means number of rats. The three loading conditions tested were unloading (Botox and steel-orthosis), mild loading (Botox), and strong loading (free cage activity).

### Gene expression 5 days post-injury

**Fig 3:**
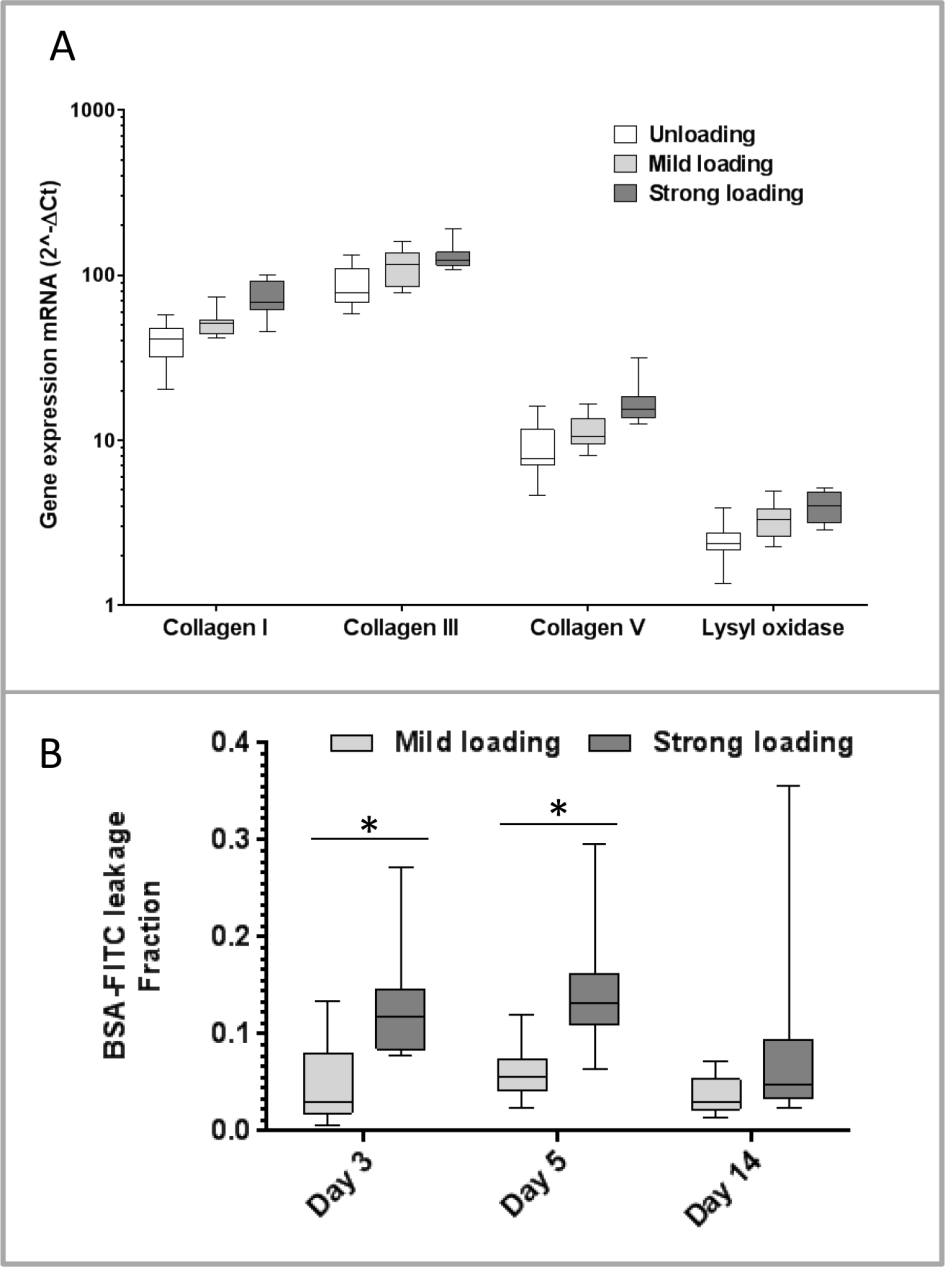
Results from gene expression and microdamage analyses. **A)** Gene expression for extracellular matrix genes, 5 days after tendon injury. The fold change of genes for *Collagen I* (*COL1A1*), *collagen III* (*COL3A1*), *collagen V* (*COL5A1*), and *lysyl oxidase* (*LOX*) are shown. Three different loading conditions were tested; unloading (Botox and steel-orthosis), mild loading (Botox), and strong loading (free cage activity), N = 11–12. **B)** Leakage of fluorescent protein (BSA-FITC) as a sign of bleeding and microdamage: 3, 5, or 14 days after tendon injury. The result describes the fraction of the fluorescence in the tendon tissue compared to the fluorescence in the blood plasma (ratio of (counts per second / mg specimen) / (counts per second / mg blood plasma)). Two different loading conditions were tested: mild (Botox) and strong loading (N = 12). The rats were intravenously injected with BSA-FITC 1 hour before euthanasia. The fluorescence detected in the tendon tissue was normalized to the tissue weight and the fluorescence detected in the blood plasma. * p < 0.001.

Compared to unloading, mild mechanical loading up-regulated the gene expression of one gene related to inflammation (*iNOS*), and several of the extracellular matrix genes (*COL1A1*, *COL3A1*, and *LOX*; Fig 3A; Table 3, S2 Dataset). Compared to mild loading, strong loading regulated most of the pro-inflammatory mediator genes studied. *IL-6* and *COX-2* were up-regulated, whereas *TNF*, *PTGES*, and *IFN-γ* were down-regulated. Strong loading also increased the expression of two extracellular matrix genes (*COL1A1* and *COL5A1*) and decreased the expression of one gene related to recruitment of leukocytes (*VCAM-1*) and the anti-inflammatory mediator *IL-10* (Fig 3A; Table 3).

**Table 3:** Gene expression, 5 days post-injury.

	From unloading to mild loading		From mild to strong loading	
Gene	FC	p	FC	p
**Pro-inflammatory mediators**				
*IL-1β*	1.12	0.557	1.15	0.548
*TNF*	1.02	0.513	**-1.73**	**< 0.001**
*PTGES*	1.30	0.128	**-1.59**	**0.024**
*IL-6*	-1.10	0.818	**3.62**	**0.001**
*PTGS2 (COX-2)*	1.43	0.163	**3.08**	**< 0.001**
*iNOS*	**3.05**	**0.025**	-1.09	0.873
*IFN-γ*	-1.14	0.659	**-1.51**	**0.009**
**Recruitment of leukocytes **				
*ICAM-1*	1.07	0.567	-1.08	0.378
*VCAM-1*	1.11	0.202	**-1.22**	**0.016**
*SELE*	1.22	0.166	1.09	0.352
*CCL7*	1.19	0.599	-1.03	0.923
*CXCL1*	1.03	0.474	1.77	0.027
*CCL2*	1.02	0.655	-1.02	0.960
**Anti-inflammatory mediators **				
*IL-10*	1.24	0.090	**-1.77**	**< 0.001**
**Extracellular matrix **				
*COL1A1*	**1.32**	**0.015**	**1.36**	**0.005**
*COL3A1*	**1.14**	**0.018**	1.23	0.131
*COL5A1*	1.38	0.061	**1.24**	**0.002**
*LOX*	**1.39**	**0.007**	1.18	0.139
*ELN*	-1.08	0.852	1.01	0.592

FC means fold change. The p-values (p) are from Student´s t-test (N = 11–12). The three loading conditions tested were unloading (Botox and steel-orthosis), mild loading (Botox), and strong loading (free cage activity). For full name description of the genes see Table 1.

### Detection of microdamage 3, 5 and 14 days post-injury

Leakage of blood plasma in the tendon, as a sign of microdamage and indicated by fluorescent protein leakage, was higher after strong mechanical loading compared to mild loading at day 3 and 5 (p < 0.001; Fig 3B, Table 4, S3 Dataset) but not at day 14 (p = 0.049).

**Table 4:** Raw data from fluorescence measurements.

	Day 3			Day 5			Day 14			Day 5 (not rinsed)		
Group	TW (mg)	FBP	FT	TW (mg)	FBP	FT	TW (mg)	FBP	FT	TW (mg)	FBP	FT
**Mild loading**	56	150144	1724	62	524743	26021	23	500051	7208	57	412968	23509
	21	515153	1722	46	539519	32887	68	466603	6654	74	439431	59279
	22	573206	15640	71	423768	20853	79	496946	11708	76	368563	55063
	69	485686	8409	65	525270	24255	71	519494	11120	70	128344	9910
	87	341884	13515	78	515541	19741	9	494967	4957	73	442932	37154
	21	541243	3992	57	467031	26978	80	500913	22064	82	58824	6806
	54	361568	6826	84	402111	27638	56	449473	18969	64	435657	24203
	56	442009	42086	61	50475	5495	24	495616	11062	84	394037	24530
	58	546491	34073	43	502731	7056	78	500184	14873	88	447779	38303
	58	563679	29736	73	533849	20655	60	483116	12331	104	452198	31583
	59	556197	15658	55	505247	20370	60	533389	10643			
	61	543009	8669	56	526721	17248	58	466113	11052			
**Strong loading**	78	502976	41501	93	486417	68497	186	448166	25897	89	434891	76332
	42	564250	44627	95	516665	100926	71	519683	20176	91	407461	98504
	98	521429	54811	133	549119	134616	106	476049	17304	133	444884	106006
	46	484235	76475	120	515092	50497	132	281178	15473	169	426021	182636
	39	476559	18863	147	99951	20116	65	508861	57526	110	407959	158492
	50	559787	68370	131	526646	96998	132	490797	37863	144	434830	106686
	72	503901	64732	155	459786	97854	174	478699	52756	127	427110	76358
	60	483620	49031	90	227916	34280	145	438359	40152	111	425660	121801
	82	513413	46827	152	483005	121930	59	465018	15990	162	450219	65169
	128	142806	22647	78	533819	84997	186	479544	395956	127	282017	52487
	89	452957	69754	95	526943	185254	75	522096	46730			
	114	530381	77104	88	540469	106211	209	445584	110752			

**TW** = Tendon tissue Weight (mg); **FBP** = Fluorescence in the Blood Plasma (counts/s); **FT** = Fluorescence in the Tendon (counts/s). The Blank (PBS) was subtracted from the fluorescence value of the FBP and FT value. Thereafter, the FBP value was normalized to the volume of the blood plasma that was measured and FT was normalized to the tendon tissue weight (TW) as follow: ((FT/TW) / (FBP/80)). The results become non-unit and will show the fraction of the fluorescence in the tendon tissue compared to the fluorescence in the blood plasma.

The auto-fluorescence in blood plasma and tendon tissue was low (Table 5). The experiment evaluating the effect of limb perfusion day 5 showed that rinsing the injured leg from blood reduced the fluorescence remaining in the tissue when comparing the mild loading group: rinsed vs unrinsed (Table 4). The ratio between the fluorescence in the tendon and in the blood plasma was 0.061 (0.03) for rinsed, and 0.087 (0.03) for unrinsed tendons (p = 0.044). However, there was still a significant difference between tendons with mild and strong loading, which had not received limb perfusion at day 5 (0.087 (0.03) vs 0.162 (0.06); p = 0.002).

**Table 5:** Overview of the fluorescence value measured.

	Fluorescence value measured (counts/s)		
	Mean	Minimum	Maximum
Fluorescent liquid injected (10 mg/mL BSA-FITC)	495 000	475 000	552 000
Blood plasma	451 000	50 000	573 000
Tendon tissue (rinsed)	44 000	1 700	396 000
Tendon tissue (unrinsed)	68 000	6 800	183 000
Blank (PBS)	1 100	950	1 600
Auto-fluorescence in blood plasma	2 900	1 800	4 100
Auto-fluorescence in tendon tissue	3 000	2 200	3 400

The rats were intravenously injected with 1 mL of BSA-FITC (10 mg/mL) 1 hour before euthanasia (*Fluorescent liquid injected*). Blood was collected 30 minutes after the injection (*Blood plasma*). The limb was perfused with saline to clear the vessels from fluorescent protein before euthanasia and the rinsed tendon tissue was collected (*Tendon tissue (rinsed)*). To evaluate the effect of limb perfusion, 20 rats were unrinsed (*Tendon tissue (unrinsed)*). The blood plasma and the homogenized tendon tissue were dissolved in PBS before measuring (*Blank (PBS)*). Possible auto fluorescence was evaluated in 6 rats for both blood plasma (*Auto-fluorescence in blood plasma*) and tendon tissue (*Auto-fluorescence in tendon tissue*).

## Discussion

As we expected, even small changes in mechanical stimulation can improve tendon healing by increasing mechanical and material properties. Moreover, stronger mechanical stimulation increased these properties further as well as callus transverse area. Additionally, this loading condition, in contrast to mild, induced small tissue damages seen by an increased blood leakage and increased inflammatory gene expression. This suggest that the positive effect of loading derives partly from different mechanisms depending on the degree of loading.

### Mild mechanical stimulation improves the quality of the healing tendon

Mild mechanical stimulation has previously been shown to be sufficient to improve tendon healing [3]. With this new study, we could confirm this finding using a new model, a steel orthosis, to reduce load. As compared to unloading, mild mechanical stimulation increased the total strength of the healing tendon by 33%. The quality of the tissue was also improved probably due to an increased expression of collagen and other extracellular matrix genes. Patients with Achilles tendon injuries are protected from loading for several weeks by immobilization with a brace [2]. This condition corresponds most likely to the unloaded group in this experiment. The clinical rehabilitation programs, which has recently been introduced with favorable results, involves early mobilization of the injured tendon with mild loading [2, 10]. Therefore, we believe that these rehabilitation programs could correspond to the mildly loaded group in this experiment and our findings may explain the favorable results seen by early mobilization and strengthen the conclusion that this is advantageous, even with weak mechanical stimulation.

### Strong mechanical stimulation strengthens the healing tendon but also creates microdamage and affects inflammation

Strong mechanical stimulation has an even more pronounced effect and increased all structural and material properties measured. As compared to mild loading, strong loading improved tissue quality further but had a more pronounced increase on structural properties with an increase in strength and size by 200% and 62% respectively. This suggest that tissue quantity is increased by strong loading, but not by mild loading.

The fluorescent protein leakage was higher in rats with strong loading at day 3 and 5, suggesting vessel damage and blood leakage, as a sign of microdamage. As blood leakage and tissue damage can activate platelets and release alarmins, which in turn activates the inflammatory response, these results correlate to previous studies were inflammation genes were increased by strong loading at the same time-points [6, 9, 19, 20]. Additionally, there was no significant increased blood leakage 14 days post-injury, which also correlates to previous studies showing no altered inflammatory gene expression 14 days post-injury [9].

Strong loading was also associated with an altered expression of inflammation genes where mild loading had very little effect. The inflammation genes affected by strong loading were both up- and down-regulated, therefore it is difficult to say if strong loading increases the inflammatory response, as previously been shown [6, 9]. Most of the pro-inflammatory mediator genes tested were affected by strong loading. Two were up-regulated (*IL-6* and *COX-2*) and three were down-regulated (*TNF*, *PTGES* and *IFN-γ*). As the fold change of the up-regulated genes was generally higher compared to the down-regulated genes, the general response by strong loading might although be a pro-inflammatory response. This correlate with previous findings, with an immediate pro-inflammatory gene response and a prolonged increase in the M1/M2 macrophage ratio after strong loading [6, 9, 15]. The results in this study also showed a down-regulation of *IL-10* as a sign of a decrease in the anti-inflammatory response, which also corresponds to previous findings with a delay in the switch to M2 macrophages and regulatory T cells [15]. We have previously shown that strong loading increases the recruitment of leukocytes to the healing tendon, 3 days post-injury [9]. In this study, the expression of *VCAM-1*, a receptor involved in recruitment of leukocytes, was decreased 5 days post-injury [21]. This suggests that the recruitment of leukocytes is not increased at day 5 as it was at day 3. Leukocyte recruitment, due to strong loading, might have a peak during the first days after injury and thereafter decrease as the healing progress to the proliferative phase.

In summary, our results show that strong loading creates microdamage, alters the inflammatory response, and increases structural properties of the healing tendon during the first week after tendon injury. As this could only be seen in the strongly loaded group, not in the mildly loaded group, we believe that there is a correlation between these events. Microdamage possibly stimulate inflammation by chemotaxis and proliferation of both leukocytes and fibroblasts, which can increase the size of the tendon tissue [22]. A larger tendon can improve other structural properties such as the total strength, as more tissue can withstand higher forces. These results correlate to previous studies showing that damage by needling in healing tendons improves mainly the structural properties and exerts a similar gene expression pattern as short episodes of strong loading [16].

### Strong and mild mechanical stimulation might activate different mechanisms

Mild loading improve tendon healing without creating tissue damage or affecting the inflammatory response probably due to mechanotransduction mechanisms. As the quality of the tissue and the expression of extracellular matrix genes increases gradually by increased loading it suggest that strong loading also acts via mechanotransduction. However, the additional effects by strong loading, such as a more pronounced effects on the structural properties and tissue mass, might be due to microdamage which corresponds to a previous study where tendon healing could be stimulated by needling [16]. However, that study showed no large effect in material properties, while this study showed a major increase in both elastic modulus and peak stress after strong loading. This suggest that there might also be other factors, besides microdamage, that differs between strong and mild loading. Healing, during strong loading, increases the gap distance significantly compared to mild loading. An increased gap can influence tissue reorganization and tissue alignment and thereby material properties of the tendon.

In summary, our results suggest that mild mechanical stimulation improves tendon healing due to mechanotransduction mechanisms while strong mechanical stimulation improves healing due to more than one mechanism, mechanotransduction, microdamage and possibly so far unexplained mechanisms.

### Limitations and method evaluation

Rats and humans are different in many aspects, which can impair generalization. Rats are smaller, quadrupeds, and have a higher metabolic rate. The rats used in this study were also juvenile (1.5–2 months old) and therefore differs from the general patient with an Achilles tendon rupture. The age could have had an impact on neovascularization and blood vessel leakage. It is also difficult to know if it is possible to compare the different loading conditions that we use in our rat model to different loading conditions in patients. However, we believe that our unloading group corresponds to immobilization in a brace and that mild loading corresponds to early mobilization used in patients. Strong loading is quite different from human behavior as rats have evolved to avoid predation and thus do not protect injured limbs by limping as humans do.

The new method to create unloading, combining Botox and a steel-orthosis, was successful in this experiment. However, the difference between unloading and mild loading was smaller than in a previous unloading model used by Andersson, et al. where tail suspension was used instead of the orthosis [3]. One explanation for this could be passive loading of the toes, or partly loading by the gastrocnemii muscles as these muscles stretch over the knee joint as well, while the steel-orthosis was only below the knee. However, the orthosis has a lot of advantages compared to tail suspension; the rats are less stressed, their normal behavior is not affected, the steel-orthosis is more similar to human immobilization techniques, it takes less time, effort thereby allowing us to study more rats at the same time, and the orthosis is easy to remove at any time point.

Saline perfusion of the healing tendon improved precision in the fluorescence measurements, but was not required to see a difference between strong and mild loading. The auto-fluorescence evaluation showed that fluorescence was detected in blood plasma and tendon tissue but to a lesser extent compared to the BSA-FITC injected rats.

## Conclusion

We have shown that mild mechanical stimulation has a strong stimulatory effect on healing tendons without damaging the tissue or altering inflammation. As this type of loading probably corresponds to early mobilization in patients it strengthens the conclusion that early mobilization of a ruptured healing tendon is advantageous. We have also shown that strong mechanical stimulation has a stronger stimulatory effect on tendon healing but it also creates microdamages and alters the inflammatory response in association with an increased tissue mass. This degree of loading in the rat might correspond to excessive loading in humans, and might not be beneficial for the healing tendon in the long run primarily due to tissue elongation, which is seen in this study or an increased re-rupture risk.

## Supporting information

S1 Dataset Data from mechanical evaluation. Data from each rat for callus width and depth, gap distance, transverse area, peak force, peak stress, stiffness, elastic modulus, and energy uptake.
(XLSX)

S2 Dataset Data from Quantitative real-time PCR. CT values for each gene and each rat.
(XLSX)

S3 Dataset Data from detection of microdamage. Detection of leakage of BSA-FITC in the tissue and in blood. TW means tissue weight; FBP means Fluorescence in the Blood Plasma (counts/s); FT means Fluorescence in the Tendon (counts/s).
(XLSX)
